# Intervention for Smokers through New Communication Technologies: What Perceptions Do Patients and Healthcare Professionals Have? A Qualitative Study

**DOI:** 10.1371/journal.pone.0137415

**Published:** 2015-09-04

**Authors:** Jose Manuel Trujillo Gómez, Laura Díaz-Gete, Carlos Martín-Cantera, Mireia Fábregas Escurriola, Maribel Lozano Moreno, Raquel Burón Leandro, Ana María Gomez Quintero, Jose Luis Ballve, María Lourdes Clemente Jiménez, Elisa Puigdomènech Puig, Ramón Casas More, Beatriz Garcia Rueda, Marc Casajuana, Marga Méndez-Aguirre, David Garcia Bonias, Soraya Fernández Maestre, Jessica Sánchez Fondevila

**Affiliations:** 1 Centro de Salud Cuevas del Almanzora, Servicio Andaluz de Salud, Almería, Spain; 2 Primary Healthcare University Research Institute IDIAP Jordi Gol, Barcelona, Spain; 3 Centre d’Atenció Primaria La Sagrera, Institut Català de la Salut, Barcelona, Spain; 4 Centre d’Atenció Primaria Passeig de Sant Joan, Institut Català de la Salut, Barcelona, Spain; 5 Centre d’Atenció Primària La Marina, Institut Català de la Salut, Barcelona, Spain; 6 Centre d’Atenció Primaria Turo, Institut Català de la Salut, Barcelona, Spain; 7 Centre d’Atenció Primària Florida Nord, Institut Català de la Salut, Hospitalet de Llobregat, Spain; 8 Centro de Salud Santo Grial, Servicio Aragonés de Salud, Huesca, Spain; 9 Centre d’Atenció Primaria Sant Antoni, Institut Català de la Salut, Barcelona, Spain; 10 Centre d’Atenció Primaria Goretti Badia, Institut Català de la Salut, Barcelona, Spain; 11 Centre d’Atenció Primaria Vallcarca-Sant Gervasi, Institut Català de la Salut, Barcelona, Spain; The University of Auckland, NEW ZEALAND

## Abstract

**Background:**

The use of information and communication technologies (ICTs) in the health service is increasing. In spite of limitations, such as lack of time and experience, the deployment of ICTs in the healthcare system has advantages which include patient satisfaction with secure messaging, and time saving benefits and utility for patients and health professionals. ICTs may be helpful as either interventions on their own or as complementary tools to help patients stop smoking.

**Objectives:**

To gather opinions from both medical professionals and smokers about an email-based application that had been designed by our research group to help smoking cessation, and identify the advantages and disadvantages associated with interventions based on the utilization of ICTs for this purpose.

**Methods:**

A qualitative, descriptive–interpretative study with a phenomenological perspective was performed to identify and interpret the discourses of the participating smokers and primary healthcare professionals. Data were obtained through two techniques: semi-structured individual interviews and discussion groups, which were recorded and later systematically and literally transcribed together with the interviewer’s notes. Data were analyzed with the ATLAS TI 6.0 programme.

**Results:**

Seven individual interviews and four focal groups were conducted. The advantages of the application based on the email intervention designed by our research group were said to be the saving of time in consultations and ease of access for patients who found work timetables and following a programme for smoking cessation incompatible. The disadvantages were thought to be a lack of personal contact with the healthcare professional, and the possibility of cheating/ self-deception, and a greater probability of relapse on the part of the smokers.

**Conclusions:**

Both patients and healthcare professionals viewed the email-based application to help patients stop smoking as a complementary aid to face-to-face consultations. Nevertheless, ICTs could not substitute personal contact in the smoking cessation programme.

## Introduction

The use of information and communication technologies (ICTs), such as internet, mobile phones, and instant messaging services, is increasing considerably. According to the Instituto Nacional de Estadística (National Institute of Statistics) [INE, 2014], 74.4% of Spanish households have internet access and 76.2% of the population aged between 16 and 74 years were connected to it at least once during the previous 3 months. 96.4% of households have a mobile phone and 67.2% have a broadband internet connection by means of a hand-held device (latest generation mobile phones, etc.) [1].

The most recent report from the Asociación para la Investigación de Medios de Comunicación (Association for Media Research) states that Spaniards use more instant messaging services (79.5% in the previous month) than email (78.2%) [AIMC, April 2014]. In addition, the number of instant messaging services increased by 14% with respect to the same period last year whilst email decreased 2% [2]. Regarding the use of pay systems for instant messaging, Stadista, the Statistics Portal, reported that the global number of active users of WhatsApp during August 2014 was around 600 million [3]. By mid-2014, approximately 65% of mobile phone users in Spain were active on WhatsApp [4].

In a similar manner, the deployment of ICTs in the healthcare sector is increasing, mainly for appointments, computerized clinical records, electronic prescriptions, communicating test results, and searching for medical information. In spite of the fact that there are a number of limitations, including the perception that there is not enough time to devote to new technologies, lack of experience in their use, and uncertainty about the effectiveness of ICTs [5], there do appear to be some advantages. Patients have expressed satisfaction with the secure message systems, and healthcare professionals report time saving benefits. The latter consider that electronic messaging meets privacy regulations and they have not reported any negative effects. Finally, the short-term financial benefits of ICTs must be emphasized for large management groups such as hospitals and primary healthcare centers (PHCs) [6].

In 2011 a WHO report on e-Health indicated a lack of policy implementation with respect to this issue and highlighted a general perception of elevated costs and the need for regulation. In contrast, ICTs were well-received by patients who considered them to be cost-effective [7].

Hsiung et al. reviewed the impact of computer-based therapies (e-therapy) on a number of situations and observed that follow-up by email is useful, provided that it is preceded by direct and personal contact with the patient [8]. The utility of including internet as a tool in the process of tobacco cessation is becoming increasingly evident, given the necessity of reaching more of the smoking population irrespective of their ethnic or cultural background [9].

The profile of individuals searching internet for help in smoking cessation is made up mainly of women (59%) and young people. In general, they intend to stop smoking (53%) and are looking for information about how to do this and possible medication. There are also those who have stopped and are seeking for ways of how to cope with abstinence [10].

The 2013 updated Cochrane Review on internet-based interventions for smoking cessation reported that interactive and more personalized interventions had a higher rate of success than passive methods (17% over 6 months). In addition, results suggested that some internet-based interventions could assist smoking cessation at six months or longer, particularly those which were interactive and tailored to individuals. However, further research is needed [11].

Various qualitative studies have been carried out to identify barriers and facilitate elements that influence communication between the general practitioner and the smoker [12], and to describe the difficulties in dealing with smoking addiction in some population groups [13]. Frisby et al. in their study on why individuals consulted WebPages in order to stop smoking reported time saving and anonymity as two of the principle motives [14].

Research into interventions based on the use of ICTs is augmenting progressively, although specific ICT-based healthcare interventions for smoking cessation remain to be developed. Moreover, to the best of our knowledge, few studies have, analyzed the attitudes of both patients and healthcare professionals with respect to the design and use of ICT-based interventions. The aim of the present study was to examine in depth at the opinions of smokers attended in PHCs, and their health professionals, in order to gain understanding about the barriers and facilitators of ICT use in a health environment and, in particular, for smoking cessation. In addition, the results of the present study facilitated the design of an email-based application created by our research group (TABATIC) to help smokers quit [15].

## Materials and Methods

### Design

A descriptive–interpretative qualitative study was carried out from a phenomenological perspective to identify and interpret the discourses of healthcare professionals and smokers attended in PHCs with respect to the advantages and disadvantages of the use of ICTs for smoking cessation. This kind of methodology was selected as being the most suitable to assess the opinions, expectations, attitudes, and perceptions of the patients and professionals with respect to the issue. It also allowed the study to be carried out within the context of a number of PHCs employing diverse ICT tools. The present study was part of the project: "Effectiveness of an intensive E-mail based intervention in smoking cessation (TABATIC)" funded by the Spanish government (Fondo de Investigación Sanitaria (PI11/00817) [15] and complements the results of another study which aimed to quantitatively describe the use of ICTs among a population of smokers attended in primary care [16]. The results of this qualitative study were fundamental in designing the e-mail based intervention of the TABATIC clinical trial.

### Participants

Study participants included both smokers attended in primary care and health professionals from 8 PHCs in Barcelona, Spain.

#### Smokers

Inclusion criteria for smokers were: a) aged: 18–75 years; b) smoking at least one cigarette per day; and c) regularly attending the PHCs. Subjects suffering from terminal illnesses or severe psychiatric disorders, not understanding Spanish or Catalan, or not having signed the informed consent were excluded. We selected a convenience sample from those patients who visited their health professional for whatever reason, fulfilled the inclusion criteria, agreed to participate, and signed the informed consent. In order to guarantee diversity on the discourses concerning ICT use and applications in smoking cessation interventions we selected participants of both sexes, of different ages, and with varying levels of ICT use. The total number of smokers included in the study was 11 and was determined by information saturation (2 individual interviews and 2 focal groups). Classification criterion for smokers were based on a previous study undertaken in a similar population which showed that the factors positively associated with ICT use were sex and age (mainly <45 years old) [16]. Smokers were, therefore, classified into groups according to gender, age (18–45 and ≥45), and ICT use (mid-high frequency > once a week; low frequency ≤ once a week) which, in this case, coincided with age (mid-high frequency 18–45 years/low frequency ≥45 years) as reported in a previous study [16].

#### Primary health professionals

General practitioners and nurses were recruited among those participating in the Primary Care Without Smoke Programme (*Programa de Atenció Primària Sense Fum)* and interviewed individually. To facilitate recruitment, possible participants were also enrolled through the so-called snowball technique at the Unitat de Suport a la Recerca- IDIAP Jordi Gol (Research Support Units of the University Institute for Primary Care Research Jordi Gol) and the tobacco addiction group of the Societat Catalana de Medicina Familiar (Catalan Society of Family Medicine [GRAPAT]). Healthcare professionals were classified into two groups according to their professional status (general practitioner or nurse) and self-reported ICT (email, social networks, mobile phone and/or videoconferencing) use. Primary health professionals responded to the following question: With which frequency do you use the new ICTs? Possible answers were: mid-high frequency (habitual use) or low frequency (low use or only for professional reasons). The health professionals were then classified into “mid-high frequency use” and “low frequency use” groups.

### Ethics statement

The study protocol was approved by the Clinical Research Ethics Committee of the Jordi Gol Research Institute for Primary Care. Written informed consent was obtained from all patients prior to participation. The study was performed in accordance with the declaration of Helsinki II [17].

### Data collection methods

The following data collection methods were employed.

**Semi-structured individual interviews with smokers and healthcare professionals:** Both open and guided questions were posed, aiming to gather and interpret opinions about the use of ICTs in the treatment of health problems in general, and in dealing with tobacco addiction in particular. The interviews also tried to identify possible barriers, limitations, and applications, and to identify participants’ perceptions of such issues as habitual and potential behavior (Table 1).

**Table 1:** Individual interviews.

**Individual healthcare professional interviews (ages)**		
Professionals with mid-high frequency use of ITCs	Two nurses: female (42/52)	
Professionals with low frequency use of ICTs	One nurse: female (59) and two general practitioners: female (39/50)	
**Individual patient interviews (ages)**		
Patient (<45 years)	One female (26)	Mid- high frequency use of ICT
Patient (≥45 years)	One female (51)	Low frequency use of ICT

**Focal groups with smokers and healthcare professionals:** Group interviews with patients and healthcare professionals were also held. Each group analyzed the use of different ICT intervention strategies (websites, emails, mobile telephones, and mixed methods). After the presentation of the strategies, the participants were asked their opinions on the limitations and utility of each one with respect to treating tobacco addiction.

In total, seven individual interviews were conducted (five with healthcare professionals and two with smokers) and four focal groups (two groups of patients and two of healthcare professionals) (Table 2).

**Table 2:** Focal group interviews.

**Healthcare professional focal groups (ages)**		
	**Number of participants**	**Participants’ profile**
Professionals with high frequency	4	Nurses: two females (52/29)
use of ICTs		General practitioners: one female (54) and one male (49)
Professionals with low frequency	3	Nurses: two females (57/42)
use of ICTs		General practitioners: one female (43)
**Smoker focal groups (ages)**		
	**Number of participants**	**Participants’ profile**
Patient (≥ 45 years)	5	Two females (46/57) and three males(61/61/64)
Patient (< 45 years)	4	Three females (32/37/44) and one male (43)

### Fieldwork

Participant recruitment and group/individual interviews took place between June and December 2012. All interviews were carried out at the headquarters of the CAMFIC (Societat Catalana de Medicina Familiar i Comunitària [Catalan Society of Family and Community Medicine]), Barcelona, Spain. Group interviews were moderated by an expert in qualitative investigation and included an observer; both were unfamiliar to the participants. Individual interviews lasted between 15 and 30 minutes, and focal group meetings about 90 minutes.

### Analysis

Both group and individual interviews were audio recorded with the participants’ consent. A systematic and literal transcription was made, with notes from the interviewers, for later analysis. To simplify data analysis, the ATLAS TI 6.0 programme was used.

First, a careful transcription reading was made and the text then split up into meaningful information units. These units were coded following a mixed strategy (emerging and predefined codes according to the study objectives), and categories were developed on the basis of grouping codes with the same theme and those defined by the SWOT technique (strengths, weaknesses, opportunities, and threats). This was followed by the creation of a hypothesis and the grouping and interpretation of data within a theoretical framework. The hypotheses that emerged were: a) participants (smokers and health professionals) considered that ICT-based interventions provide assistance for smokers to quit and are complementary to interactions and face-to-face visits between patients and primary health professionals, and b) contributions of the present study can be of great utility to design an ICT-based intervention appropriate to the context and well accepted by patients and health professionals.

Finally, the points of agreement and disagreement were analyzed and triangulation of the results performed by an interview moderator and an expert analyst. In addition, a reflexive verification of the results was carried out by the research team.

## Results

### Participants’ characteristics

Fourteen smokers attended in primary care and 14 health professionals were invited to take part in the study. The final number of participants was 23 (82.1%): 11 smokers (4 men and 7 women, mean (SD) age: 47.45 years (12.59)) and 12 health professionals (1 man and 11 women, mean (SD) age: 47.33 years (8.57)).

Results are divided into healthcare professionals and smokers.

#### Healthcare professionals

Participants were categorized into two profiles:

- **Mid-high frequency ICT users:** participants believed that an email application to help smoking cessation could be very useful and gave it a high rating.
- **Low frequency ICT users:** they were skeptical about ICT interventions for smoking cessation, particularly when used alone.

#### Smokers

Participants were classified as:

**Patients aged 18–45 years:** they frequently use ICTs and rate these technologies highly.
**Patients aged ≥ 45 years:** they also habitually use ICTs and value them highly. In general, this age group had little interest in giving up smoking at present although considered it a future possibility.

A list of additional interview excerpts can be accessed in the supplementary materials [see S1 Text]

Opinions and beliefs of the participants in the study, both health professionals and smokers, were then expanded in terms of the items studied: ICT use in personal life, in health care, and in smoking cessation, and opinions and evaluations regarding the future design of an email-based application to help patients stop smoking.

### 1. ICT use and evaluation in personal life

**Healthcare professionals** considered that the habitual use of ICTs such as the mobile telephone (SMS, WhatsApp, etc.), email, and social networks was integrated into daily life at varying levels (work and personal).

They differentiated between the personal and professional employment of ICTs (e.g. SMS are more personal than emails) and evaluated them as being easy to use, rapid, and economical.

**HP3:** *"I think that we have become familiarized*, *it is one of our daily habits*, *in the way we communicate; I don’t know how we did it before…"*

According to **smokers**, they habitually used ICTs such as the mobile telephone (short message service (SMS) and WhatsApp), email, and social networks. In the two age groups variations were found for the level and use of ICTs. The older group claimed to have less knowledge, interest, and time and mainly employed SMS on their mobile telephones and connected to the Internet via a computer. The younger group had SMS and computer connections plus Twitter, Facebook, WhatsApp and the like integrated into their mobile telephones.

Overall, they considered ITCs to be very useful, as an economical and rapid means of communication and information.

With respect to disadvantages, patients thought that the ICTs evolved very quickly and generated a strong dependence, particularly in young people. They also had an impact in that they represented a considerable problem if they failed and there was a risk of de-personalization in social relationships.

**PA10:** *"Because the truth is that*, *I don’t know*, *it’s something that*, *day to day*, *you know*, *or rather*, *you’re up to date*, *you’re informed about everything*. *For example*, *I don’t know*, *the bus timetable app you use almost every day as well*. *Or…I don’t know*, *there are various apps that…*.*well…that you use all through the day*. *(…) Well*, *let’s see*, *advantages*, *that’s what I say*, *no*? *They’re very useful*. *You have a variety of options to help you from day to day*.*"*

### 2. Use of ICTs in health care

Internal email and web consultations were the two ICTs most frequently used by **health professionals** at work. To a lesser extent, ICTs were also employed to communicate with patients, with a rapid increase in SMS use, although phone calls were preferred and Skype had been proposed. Depending on the business management company and the professional status of the participants the deployment of ICTs varied. Nurses used email to communicate internally and SMS to contact patients; family doctors used email for internal communication and were starting to employ them with patients who had previously authorized their use.

Healthcare professionals believed that time was needed for ICTs to play an appropriate role in the working environment. In addition, the legal aspects had to be taken into consideration.

The following advantages and disadvantages were observed:

Whether or not they were interested in ICTs, healthcare professionals were of the opinion that they represented new challenges and opportunities. As a complement to personal consultations ICTs could facilitate communication and save time. The main drawback was diminished direct contact with the patient. Other disadvantages on the part of the healthcare professionals included lack of time and technology to achieve optimum results from ICTs, unequal access, and insufficient motivation.

**HP6:** *"And I always have it to hand*, *because at any given moment you could be at home and you see it*. *Because at any moment I could be sent a message on my mobile*, *“I have gone to this house and found this*, *what should I do*?*” Well if you do it by email you’re not always there and by mobile it’s in a moment*, *“Well listen*, *do this*, *do that*, *and I’ll be there*.*” Obviously this is immediate because we always carry it with us*.*"*

For **smokers** unequal access to the new technologies (taking into account age, cultural level, and socio-economic situation), and a possible negative perception (ICTs being a substitute for healthcare professionals on the grounds of cost) represented some downsides. In smokers, differences appeared according to age. The patients knew via the website about some applications for health care (visiting hours, accessing test results, etc.) with internet being the most common tool for making appointments (mainly young people). The use of ICTs (especially email) to receive test results was valued although they often had to be explained. The smokers mentioned that ICTs could save time and facilitate access to medical services by eliminating the need for displacement or loss of time spent waiting. On the other hand, ICTs could result in the loss of personal contact and patients preferred personal visits to the surgery.

**PA10:** *"Yes*, *well*, *basically what I do a lot is ask for an appointment by internet*. *What’s happening is*…*well*…*it’s not the same*, *no*? *By internet means that you sit in front of a computer*, *click on the hour*, *good*, *put in your details and so on*, *click on the hour that suits you*, *and …now you have an appointment set up*, *don´t you*? *Mmmm*, *it’s what I tell you*, *let’s see… it’s good*, *because you don’t have to queue or do anything*, *it’s good*. *But then*, *on the level…what we said---dealing with the professional*, *well that also changes a little*. *But*, *at the level of getting an appointment*, *it seems good to me"*

### 3. Use of ICTs in smoking cessation

In general, **health professionals** were not aware of the utility of ICTs in the field of smoking cessation with the exception of telephone calls. Their opinion was that personal consultations were more motivating and that the ideal situation would be a combination of personal consultations and ICTs.

The application of ICTs for smoking cessation could have advantages such as saving time, immediacy, wide-spread use, and providing extra motivation for the patient. Disadvantages included the lack of real motivation on the part of the patients which could be exacerbated by not having to commit themselves to travelling to the surgery, and the time healthcare professionals would need to become familiar with the ICTs. The latter were also dubious as to whether the required financial investment would be forthcoming and the infrastructure prove adequate.

Healthcare professionals valued the time and effort they had invested in the consultations and envisaged a reduction of contact between themselves and the patients (de-personalization).

**HP9:** *"Well*, *starting off*, *I find them…*. *Let’s see*, *stopping smoking isn’t easy*, *people need a lot of support and the new technologies…*, *to substitute for a personal visit*, *I don’t see that at all clearly*. *Now*, *as support*, *yes*. *If as well as the personal visit*, *you keep on being reminded*, *yes*, *but as a substitute no*.*"*

**Smokers** were unaware of ICTs related to smoking cessation and considered that the most effective way to stop was self-motivation and conviction combined with personal consultations with healthcare professionals.

**PA9:** *" It’s that when you enter into anxiety*, *it’s seconds*, *I speak from experience*. *In those seconds*, *that is*, *they’re few*, *but you cannot cut it off and say…*.*Clearly it’s strength of will*, *but I don’t see myself saying “right now I am anxious*, *I’m going to*…*”(…) No*, *I won’t do it*, *because even though I have done it*, *I have the experience that yes I can suppress it or for seconds I’m not going to start writing that this is happening*, *if this is happening to me*. *It’s that at any moment I can light a cigarette and I’m on top of it…"*

### 4. Design of an email-based application to help patients stop smoking

**Health professionals** believed that personal consultations could be complemented by an email-based application although it had to be very clear to whom it was directed.

Also to be taken into account were the internal organization of the centers, the peculiarities of the process of smoking cessation, and coordination among healthcare professionals. Other proposals included additional personal consultations between the messages being sent and individual websites (e.g. blogs where children could comment, the equation of the price of tobacco with hobbies could be calculated etc.). The application permits personalized feedback and is intended for use in the nurses’ consulting rooms. Other measures such as sending positive support messages (original ones, not the typical “Well done, carry on…”) or establishing a forum/ chat room moderated by professionals and which could promote interaction among patients, were suggested.

Some proposals were made with respect to follow-up, particularly at commencement of the cessation programme, for instance, some PHC consultations could be substituted by SMS/email on a more frequent basis. Moreover, the patients should be asked whether they prefer SMS or email even though the former is considered more personal.

The needs of the healthcare professionals when using the application depend on the time dedicated to it, the motivation of their colleagues, training, and the availability of new technology resources in their work environment.

**HP6:** *"For me*, *as a single tool*, *no*, *personally*, *ah*, *as a smoker*, *no*. *It’s not just receiving a message*, *which is very easy to erase*. *It has to be something that…everything helps*, *right now*, *but as a single tool*, *I don’t know…"*

**Smokers** believed that emails would help as a complement to personal PHC visits. In the first phases of follow-up patients preferred personal meetings, and later contact by email or SMS. They considered this an innovative tool which was motivating and time saving, and, at the same time, provided immediacy to professional access.

As to disadvantages, patients saw a loss of personal contact with healthcare professionals and admitted that the possibility of cheating/self-deception would make it easier to relapse. They proposed that the application serve a complementary role and have other functions (somewhere to leave comments and feedback, a forum to share experiences, etc.). Patients preferred to receive an SMS or WhatsApp and reminder messages when they had stopped smoking.

The needs of the patients with respect to the application would be to evaluate the use of medical support (pills or patches), the availability of such resources as ICTs, and the will power to give up.

**PA2:** *"I think that yes*, *because once in a while some reminder is needed*, *I believe that’s fundamental*. *It’s as if you were a second division referee*, *you’re not alone; you feel very much alone and then you receive the messages*. *It’s how do you say “keep going forward”*. *I understand that this could be complementary; also you have to recognise that if there is no will there is nothing*.*"*

## Discussion

### Analysis of data: ITCs and Health

All the informants, to a greater or lesser degree, employed new technologies in their daily lives. In concurrence with other studies, there were differences between older and younger individuals, with the latter using more and newer applications [16, 18]. The most common ICTs were those found on mobile telephones (SMS/WhatsApp), email, and websites. Evaluation of the new technologies in general was positive (e.g. they provide communication with loved ones and make life easier) with the exception of one participant who gave a very negative evaluation, possibly due to lack of knowledge and habitual use. Some informants, however, commented that there might at times be the sensation of being controlled.

In the health field, patients were aware of the websites to make appointments, and those who employed them were mainly the younger ones. Our results, however, coincide with recent literature in that both the use and knowledge of ICTs in healthcare is less than could be hoped for [19]. Our study shows that the notification of appointments and test results by SMS was positively rated. Nevertheless, a drawback of the latter is that a medical professional is needed to interpret results correctly. Both participants and healthcare professionals commented that the use of ICTs simplified access to information related to health aspects. This could have a negative aspect, above all for hypochondriacs: excess of information might lead to obsessiveness and the creation of imaginary conditions. Another study reported that most patients had no problem with physicians using mobile devices to inform themselves about the patients’ condition, and the vast majority of patients did not object to physicians using their devices for patient education and providing or illustrating information [18]. A qualitative study showed that physicians saw a benefit in using email with specific patients in specific situations and reported better communication with those who had chronic disease and required small, frequent changes in management. Other advantages included the continuity of communication with patients, the ability to respond to non-urgent issues in their own time, and improved efficiency in certain scenarios [20]. The uncertainty of involving office staff, and an increased workload for the physicians, were perceived as potential drawbacks [20]. These data are similar to those found in our study, where patients indicated that the use of ICTs facilitated access to the health system and diagnostic test results.

Koehler et al. concluded that it was possible that attitudes towards internet were more favorable than those for applications because the former had been available for a longer time and individuals were more familiar with it. Prior to using newer electronic resources, especially applications, medical professionals should inform patients with regard to their intended use to avoid potential misconceptions. This study also showed that most participants were unaware of medical applications designed for the general public, coinciding with our results [21]. Nevertheless, other authors have reported that one third of patients would like to be able to contact their doctors using ICTs [18]. These authors observed that differences in the way patients and doctors perceive the use of mobile devices could be attributed to age and level of education, and, in a professional context, excess time to familiarize oneself with mobile technology and lack of interest were not negative contributing factors [18].

A Cochrane Database of Systematic Reviews conducted by Atherton et al. analyzed email for clinical communication between patients/caregivers and health care professionals. It showed, among other results, that the perception of email use by healthcare professionals and patients was more positive in those that were actually employing email than in those being asked to consider using it. Additionally, it recommended that qualitative research methods could be utilized to explore the factors that are important for patients and healthcare professionals [22].

### Use of ITCs in helping smoking cessation

Our study shows that patients were unaware of smoking cessation ICTs and did not mention anything with respect to them. Those who had tried to stop smoking at some time had done so either with medical assistance or on their own with help from books. Patients had no confidence in on-line smoking cessation support. Professional medical help was valued highly, particularly if the patient was strongly self-motivated, although even under these circumstances they considered the personal support of a healthcare professional essential. The health professionals consulted had similar beliefs, but differed in that, irrespective of the method used, the principal issue in smoking cessation was to be aware, motivated, and convinced.

A number of studies have assessed the effects of ICT-based interventions. Te Poel et al. evaluated the efficacy of a computer-tailored smoking cessation intervention with email [23], Polosa et al. demonstrated the utility of email consultation messages in a smoking-cessation program although the number of included patients was somewhat low [24]; and Lenert et al. determined the effectiveness of an automated, educational email messaging system individually received within the framework of a smoking cessation intervention [25]. Also, increased smoking abstinence and reduced frequency of smoking was observed in young adults after online peer support via email [26], and in adolescents after a home-based internet intervention [27, 28]. Despite these studies, there is little information about the opinion of smoker participants regarding ICT-based interventions.

### Opinions regarding an email-based application to help patients stop smoking

In concurrence with other studies on the possible values of ICTs to complement smoking cessation, the healthcare professionals considered that an application based on email would provide valuable support to existing interventions [14]. They believed that receiving messages at key moments during the process of smoking cessation would be beneficial in maintaining patients’ motivation. In addition, they considered it positive to establish a space where the patients could regularly note their feelings. In another qualitative study, this time performed with pregnant smokers who completed the intervention arm of a pilot, randomized, controlled trial of a novel internet-based smoking cessation intervention, similar ideas were expressed. These included the notion that the application should offer novelty, particularly in the case of long-term support, employing an interactive environment which offered attention-grabbing features that could be explored regularly and engaged with to facilitate craving management [29].

With respect to follow-up, patients believe assistance depends on the stage of the process they are going through and how they are coping. If it is near the beginning, and the patients are suffering periods of anxiety, face-to-face meetings are essential. As more advanced stages of change are reached and the patients become stable, phone calls or SMS messages would be useful. Patients expressed preference for the latter as they are more accessible and immediate. They believed that a face-to-face meeting with a medical professional was very important, particularly when breaking the tobacco habit. They commented that smoking cessation was hard even with personal help so that at a distance, employing ICTs which implied loss of contact, it would be even more difficult and a relapse might be more probable. Moreover, with ICTs it would be easier to fool both oneself and the healthcare professional involved in the follow-up process. In contrast, Herbec et al. described mixed views towards receiving regular emails. Some individuals saw automated emails as impersonal or intrusive and akin to spam, others preferred extensive email support containing additional advice, encouragement, and information. They reported that regular emails helped them to remain engaged with the website, maintain motivation, and remind them not to smoke [29].

Smoker participants in our study proposed that an email-based application to help patients stop smoking should include some specific functions including a site to leave comments at any time, the possibility of receiving feedback on their results (e.g. days without smoking, overcoming a relapse, etc.) or an open space/ forum in which different application users could share their experiences. In this way the application could function as a type of on-line group therapy. This has been commented on in other studies where pregnant smokers were interested in motivating each other with personal stories and experiences about smoking cessation [29].

Smoker participants in our study also asked about how often messages were to be sent and whether the cessation process using the email-based application would involve chewing-gum, patches and other aids in a similar manner to face-to-face interventions. To establish and reinforce the commitment and enthusiasm of the patients who intended to stop smoking using the email-based application, smokers proposed that a space be established in which a summary could be written at any moment about the on-going process. Patients would be responsible for giving feedback to the doctor about how they felt. Additionally, messages from the physicians would give positive support, especially at key moments of the process, which would help to maintain commitment. In the qualitative study conducted by Herbec et al. participants expressed a strong need for help with craving management and believed that the application could encourage them to remain abstinent by offering a distraction [29]. They were interested in interactive, self-monitoring tools for tracking progress and documenting benefits, such as charts and personalized feedback on the number of smoke-free days, money saved, and health gains. The majority expressed a need for frequent positive support and encouragement, either via the website or emails, ideas that were also put forward in our study [29].

An interesting proposal during our research came from a patient who on one occasion had given up smoking, but had started again. He considered that the problem was that he did not have anyone to help him stay off tobacco. He believed that once the goal of stopping smoking has been reached, the application should continue sending reminder messages of the type, “Remember you mustn’t smoke” or something similar to avoid a relapse. In the study of Herbec et al. it was emphasized that assistance with failed cessation attempts should be offered alongside support for first-time attempts. Participants felt encouraged by establishing communication with other pregnant women who were using the same smoking cessation application [29].

The majority of our smokers did not believe that they could stop smoking without pills or patches. They appreciated having new technologies available, above all in the case of older patients, who in many cases lacked the necessary resources to be able to use them. These patients were of the opinion that stopping smoking was no easy task and that they would need more will power if using an email-based application cessation rather than face-to-face consultations.

Flym et al.[19] assessed the expectations and experiences of patients and staff about a new eHealth (for booking patient appointments) service. When asked about future eHealth facilities related with the application, most patients were not interested in using email to exchange information with their general practitioners, and a third said that they would prefer to talk to doctors rather than send emails. General practitioners showed more positive support for email contact than was expressed by patients, although a number expressed concerns about confidentiality and the possibility of receiving abusive messages. This study showed the patients’ positive perceptions of the new eHealth service as an easier form of communication with the medical practice, and negative ones such as decreased human contact, preference for conventional use, and lack of experience with ICTs and internet [19], results supported by other studies and which coincide with our findings [5].

In the study by Herbec et al. pregnant smokers viewed the application as a potentially helpful alternative to medication, face-to-face consultations, and telephone support. They mentioned advantages such as privacy, convenience, flexibility, and constant availability. ICTs were believed to be especially helpful where traditional support was limited, unavailable or inaccessible. Other reasons for ICT acceptance were personal preferences for giving up without assistance from others, dislike of group therapy, and expecting difficulties or embarrassment with face-to-face support [29].

In summary, the main advantage reported by the healthcare professionals was time-saving in the surgery and, for the smokers, having access to a program for smoking cessation that did not interfere with work hours, normally a problem with PHC workshops, and the avoidance of unnecessary journeys to the medical centre.

With respect to disadvantages, and in agreement with other studies, one mentioned by the healthcare professionals was the lack of time available in the surgery to dedicate to ICT-based activities [5]. In the case of the smokers it might be thought that the concurrent investment in ICTs and the reduction in personnel, thus jeopardizing personal contact, were being proposed as a response to the current economic crisis. Another drawback reported was that the level of commitment and the final success of smoking cessation might be affected by the loss of contact with a healthcare professional.

### Study limitations

There are limitations inherent to the design of the study. One of the possible biases to consider is the distortion or slant of the information provided to the interviewer, and the opinion of the rest of the participants (in group work). Similarly, it is possible that the composition of the questions conditioned responses. With the aim of limiting these possible biases, the interview script was written by various researchers with different educational backgrounds and revised by an external analyst, and the interviewer had high interviewing skills. Another limitation is the fact that most of the smokers and healthcare professionals were women (corresponding with the Spanish health profile where the majority of the nurses, and increasingly physicians, are women) [30]. In addition, there were other factors such as incompatibility of work hours and interview times, family responsibilities (which limited availability for many people), and the difficulties of finding a common time for everyone. This led to a somewhat low number of participants in the interviews and focal groups; however, with the participants included we were able to arrive at information saturation. Our work complements and reinforces existing knowledge on this subject and the results could be extrapolated to another study population.

## Conclusions

Both healthcare professionals and patients saw an ICT-based application to assist smoking cessation as a complement to personal consultations which they considered to be a non-substitutable option in health care, and even more so in the case of smoking cessation. It was proposed that a greater number of personal consultations be interspersed with SMS/email messages. It should be taken into account that whoever is offered the application should be able to use it, that is to say, having both the appropriate resources and understanding of new technologies.

Coordination among healthcare professionals should be encouraged. Moreover, there should be someone responsible for supplying feedback to the patient using the application.

## Supporting Information

S1 Text Additional interview excerpts. Here are reflected excerpts of the discussions held by patients and health professionals during interviews and focus groups divided into sections and themes.
(DOCX)
