# Supplementary material for: Intervention for Smokers through New Communication Technologies: What Perceptions Do Patients and Healthcare Professionals Have? A Qualitative Study
Source: PLoS One. 2015 Sep 4;10(9):e0137415. doi: 10.1371/journal.pone.0137415 (PMC4560416; doi:10.1371/journal.pone.0137415)
Supplement: S1 Text — Here are reflected excerpts of the discussions held by patients and health professionals during interviews and focus groups divided into sections and themes. (DOCX) [file pone.0137415.s001.docx]

# Intervention for smokers through new communication technologies: What perceptions do patients and healthcare professionals have? A qualitative study

**Additional File 1: Additional interview excerpts**

Jose Manuel Trujillo Gómez **^1,2¶*^**, Laura Díaz-Gete **^3¶^**, Carlos Martín-Cantera **^2,4¶^**, Mireia Fábregas Escurriola **^5^**, Maribel Lozano Moreno **^4^**, Raquel Buron Leandro **^6^**, Ana Maria Gomez Quintero **^6^**, Jose Luis Ballve **^7^**, Mª Lourdes Clemente Jiménez **^8^**, Elisa Puigdomènech Puig **^2^**, Ramón Casas More **^9^**, Beatriz Garcia Rueda **^10^**, Marc Casajuana **^2^**, Marga Méndez-Aguirre **^11^**, David Garcia Bonias **^11^**, Soraya Fernández Maestre **^3^**, Jessica Sánchez Fondevila **^2^**.

**1.** Centro de Salud Cuevas del Almanzora, Servicio Andaluz de Salud, Almería, España. **2.** Primary Healthcare University Research Institute IDIAP Jordi Gol, Barcelona, Spain. **3.** Centre d’Atenció Primaria La Sagrera, Institut Català de la Salut, Barcelona, Spain. **4.** Centre d’Atenció Primaria Passeig de Sant Joan, Institut Català de la Salut Barcelona, Spain. **5.** Centre d’Atenció Primària La Marina, Institut Català de la Salut, Barcelona, Spain. **6.** Centre d’Atenció Primaria Turo, Institut Català de la Salut, Barcelona, Spain. **7.** Centre d’Atenció Primària Florida Nord, Institut Català de la Salut, Hospitalet de Llobregat, Spain. **8.** Centro de Salud Santo Grial, Servicio Aragonés de Salud, Huesca, Spain. **9.** Centre d’Atenció Primaria Sant Antoni, Institut Català de la Salut, Barcelona, Spain. **10.** Centre d’Atenció Primaria Goretti Badia, Institut Català de la Salut Barcelona, Spain. **11.** Centre d’Atenció Primaria Vallcarca-Sant Gervasi, Institut Català de la Salut, Barcelona, Spain.

¶ These Authors contributed equally to this work

*Corresponding author: aplaceinheaven@hotmail.com

| Theme 1: Use and evaluation of ICTs in personal life | | |
| --- | --- | --- |
| Participants | | Example interview excerpts |
| **Health Professional** | | **HP3:** "I think that we have become familiarized, it is one of our daily habits, in the way we communicate; I don’t know how we did it before…" |
| **Patients that smoke** | | **PA10:** "Because the truth is that, I don’t know, it’s something that, day to day, you know, Or rather, you’re up to date, you’re informed about everything. For example, I don’t know, the bus timetable app you use almost every day as well. Or…I don’t know, there are various apps that….well…that you use all through the day. (…) Well, let’s see, advantages, that’s what I say, no? They’re very useful. You have a variety of options to help you from day to day." |
|  |  | **PA2:** "I believe that it’s all been said, I insist, in the sense that everything goes so quickly that, for people who use them it must be…., they have an open mind. I’m not knowledgeable, but I recognise that more than the new technologies, each week there’s a new application and there are some things that you have to do “live”. I mean that for me they have surpassed me, my children are eating and they’re on their iPhone, but anyone that you see in the street, at the bus stop, they’re all the same, the whole world is doing this, and this is what bothers me." |
| Theme 2: Use of ICTs in health care | | |
| Participants | | Example interview excerpts |
| **Health Professional** | | **HP3:** "In this case I don’t need to get information from the people, I’m more of an information receiver (…) and then, with some patients, I send them an email if they want me to send them some test results or…, but I do it on my own initiative, not with the company that is …, I suppose that it’s not correct." |
|  |  | **HP6:** "And I always have it to hand, because at any given moment you could be at home and you see it. Because at any moment I could be sent a message on my mobile, “I have gone to this house and found this, what should I do?” Well if you do it by email you’re not always there and by mobile it’s in a moment, “Well listen, do this, do that, and I’ll be there.” Obviously this is immediate because we always carry it with us." |
|  |  | **HP8:** "Face-to-face visits we can sometimes do them regularly, but if the user by means of the web, a blog or an application that can reinforce this, (…) it should be available, shouldn´t it? The content, the information, the support that is needed in a specific moment, well that is the ideal complement between personal attention and non-personal attention, what we really should guarantee is continued assistance, shouldn´t we? This is fundamental if we are speaking about education and therefore for a change…ah…in habits, aren´t we? Or changes in the behaviour of people so that they acquire healthy habits, and therefore, well, on the tobacco theme I think that it is fundamental". |
| **Patients that smoke** | | **PA10:** "Yes, well, basically what I do a lot is ask for an appointment by Internet. What’s happening is...well... it’s not the same, no? By Internet means that you sit in front of a computer, click on the hour, good, put in your details and so on, click on the hour that suits you, and …now you have an appointment set up, don´t you? Mmmm, it’s what I tell you, let’s see… it’s good, because you don’t have to queue or do anything, it’s good. But then, on the level…what we said---dealing with professional, well that also changes a little. But, at the level of getting an appointment, it seems good to me." |
| Theme 3: Use of the ICTs to help stop smoking | | |
| Participants | | Example interview excerpts |
| **Health Professional** | | **HP9:** "Well, starting off, I find them…. Let’s see, stopping smoking isn’t easy, people need a lot of support and the new technologies…, to substitute for a personal visit, I don’t see that at all clearly. Now, as support, yes. If as well as the personal visit, you keep on being reminded, yes, but as a substitute no." |
|  |  | **HP3:** "Me , I believe that it will be less for you and less for them. It’s time saving for both. Depending on the situation, the moment, it could be better than a face-to-face visit". |
| **Patients that smoke** | | **PA9:** " It’s that when you enter into anxiety, it’s seconds, I speak from experience. In those seconds, that is, they’re few, but you cannot cut it off and say….Clearly it’s strength of will, but I don’t see myself saying “right now I am anxious , I’m going to...”(…) No, I won’t do it, because even though I have done it, I have the experience that yes I can supress it or for seconds I’m not going to start writing that this is happening, if this is happening to me. It’s that at any moment I can light a cigarette and I’m on top of it…" |
| Theme 4: Design of an application for smoking cessation based on an email intervention | | |
| Participants | Example interview excerpts | |
| **Health Professional** | **HP6:** "For me, as a single tool, no, personally, ah, as a smoker, no. It’s not just receiving a message, which is very easy to erase. It has to be something that…everything helps, right now, but as a single tool, I don’t know…" | |
|  | **HP11:** "Yes, the same on the patient’s side. Because you always carry it with you, it’s more convenient and quicker. But, on the professional’s side messages arrive on your mobile, and of course, you can’t get away; now you don’t have a personal life, no? On the other hand, if get an email, you open it when you are checking for work or to see what the patient is consulting you for…that’s to say, you go to work…" | |
| **Patients that smoke** | **PA2:** "I think that yes, because once in a while some reminder is needed, I believe that’s fundamental. It’s as if you were a second division referee, you’re not alone; you feel very much alone and then you receive the messages. It’s how do you say “keep going forward”. I understand that this could be complementary; also you have to recognise that if there is no will there is nothing." | |
|  | **PA11:** "There we are, nothing more, and strength of will too". | |
